# Differential expression of myosin heavy chain isoforms in cardiac segments of gnathostome vertebrates and its evolutionary implications

**DOI:** 10.1186/s12983-019-0318-9

**Published:** 2019-06-10

**Authors:** Miguel A. López-Unzu, Ana Carmen Durán, María Teresa Soto-Navarrete, Valentín Sans-Coma, Borja Fernández

**Affiliations:** 1 Departamento de Biología Animal, Facultad de Ciencias, Universidad de Málaga, 29071 Málaga, España; 2 Instituto de Biomedicina de Málaga (IBIMA), Málaga, Spain; 3 CIBERCV Enfermedades Cardiovasculares, Málaga, Spain

**Keywords:** Gnathostomes, Myocardium, Myosin heavy chain, MYH2, MYH7B

## Abstract

**Background:**

Immunohistochemical studies of hearts from the lesser spotted dogfish, *Scyliorhinus canicula* (Chondrichthyes) revealed that the pan-myosin heavy chain (pan-MyHC) antibody MF20 homogeneously labels all the myocardium, while the pan-MyHC antibody A4.1025 labels the myocardium of the inflow (sinus venosus and atrium) but not the outflow (ventricle and conus arteriosus) cardiac segments, as opposed to other vertebrates. We hypothesized that the conventional pattern of cardiac MyHC isoform distribution present in most vertebrates, i.e. MYH6 in the inflow and MYH7 in the outflow segments, has evolved from a primitive pattern that persists in Chondrichthyes. In order to test this hypothesis, we conducted protein detection techniques to identify the MyHC isoforms expressed in adult dogfish cardiac segments and to assess the pan-MyHC antibodies reactivity against the cardiac segments of representative species from different vertebrate groups.

**Results:**

Western and slot blot results confirmed the specificity of MF20 and A4.1025 for MyHC in dogfish and their differential reactivity against distinct myocardial segments. HPLC-ESI-MS/MS and ESI-Quadrupole-Orbitrap revealed abundance of MYH6 and MYH2 in the inflow and of MYH7 and MYH7B in the outflow segments. Immunoprecipitation showed higher affinity of A4.1025 for MYH2 and MYH6 than for MYH7 and almost no affinity for MYH7B. Immunohistochemistry showed that A4.1025 signals are restricted to the inflow myocardial segments of elasmobranchs, homogeneous in all myocardial segments of teleosts and acipenseriforms, and low in the ventricle of polypteriforms.

**Conclusions:**

The cardiac inflow and outflow segments of the dogfish show predominance of fast- and slow-twitch MyHC isoforms respectively, what can be considered a synapomorphy of gnathostomes. The myocardium of the dogfish contains two isomyosins (MYH2 and MYH7B) not expressed in the adult heart of other vertebrates. We propose that these isomyosins lost their function in cardiac contraction during the evolution of gnathostomes, the later acquiring a regulatory role in myogenesis through its intronic miRNA. Loss of MYH2 and MYH7B expression in the heart possibly occurred before the origin of Osteichthyes, being the latter reacquired in polypteriforms. We raise the hypothesis that the slow tonic MYH7B facilitates the peristaltic contraction of the conus arteriosus of fish with a primitive cardiac anatomical design and of the vertebrate embryo.

## Background

Myosins form a large and diverse super-family of actin-dependent motor proteins responsible for contractile movement in cells and tissues [47]. The myosin class II or conventional sarcomeric myosins constitute the group of myosins responsible for contraction in striated muscle, including the myocardium [23]. They are hexameric proteins composed of two myosin heavy chains (MyHC) and four myosin light chains (MLC) [21]. While MLC have regulatory functions, MyHC is responsible for the physical interaction with actin, producing the contraction of the sarcomere [20, 21].

Different types of muscle fibres contain distinct types of myosins, which are formed by specific MyHC isoforms or isomyosins [31]. The sequences of cardiac MyHC isoforms, especially those corresponding to the motor domain including the ATP-binding site, appear to be very similar in all vertebrates [15]. In the myocardium of adult vertebrates, two isomyosins are expressed across different cardiac segments. It has been shown that orthologs of human *myh6* are predominantly expressed in the atrial myocardium of mammals (α-MyHC), birds (aMyHC), amphibians (α-MyHC) and teleosts (aMyHC), whereas orthologs of human *myh7* are predominantly expressed in the ventricular myocardium of mammals (β-MyHC), birds (vMyHC) and teleosts (vMyHC) [4, 6, 15, 17, 34, 36, 38, 39, 49, 59, 60]. The identity and location of MyHC isoforms in adult hearts of chondrichthyans remain unknown.

Different pan-sarcomeric MyHC antibodies are routinely used to detect myosins in histological sections of myocardium, the most widely used being MF20 and A4.1025. The MF20 monoclonal antibody recognizes an epitope located in the tail domain of the MyHC [2]. Immunohistochemistry with MF20 antibodies has been extensively used to highlight the myocardium of different groups of vertebrates, such as chondrichthyans [14], teleosts [24], *Xenopus* [44], birds [45] and mammals [52]. The A4.1025 antibody is another pan-sarcomeric MyHC monoclonal antibody which reacts with an epitope near the ATP-binding site of the MyHC in a wide variety of species [7, 10]. Immunohistochemical studies with A4.1025 are less common in the literature than those with MF20, but myocardium-specific reactivity has been confirmed in a wide range of species, i.e. *Xenopus* [1, 48], mouse embryos [29], dogfish embryos [61] and zebrafish embryos [22].

In an ongoing research on the histomorphology of the chondrichthyan heart, using the lesser spotted dogfish (*Scyliorhinus canicula*) as an animal model, we have found that MF20 immunohistochemical signals show a homogeneous distribution throughout the heart, similar to that found in other vertebrates. Nevertheless, A4.1025 signals showed a heterogeneous pattern in the different cardiac segments. These preliminary results suggested that the dogfish may express distinct MyHC isoforms in a segment-specific manner. To test this hypothesis, we performed western blot analyses to demonstrate the specificity of MF20 and A4.1025 for the cardiac MyHC in the dogfish, slot blot combined with colorimetric analyses to verify segment-specific MyHC isoform expression and proteomic analyses using nano HPLC-ESI-MS/MS and ESI-Quadrupole-Orbitrap to identify and quantify the isomyosins differentially distributed in dogfish hearts. In addition, we calculated the relative affinity of the A4.1025 antibody for the cardiac MyHC isoforms by immunoprecipitation combined with protein quantification. Finally, we performed immunohistochemistry with MF20 and A4.1025 antibodies in the heart of different gnatosthome species (elasmobranchs, batoids, polypteriforms, acipenseriforms, teleosts and mammals) in order to assess the MyHC specific immunohistochemical signals in the cardiac segments of different taxa across the phylogeny.

## Results

### Anatomical and immunohistochemical findings

**Fig. 1:**
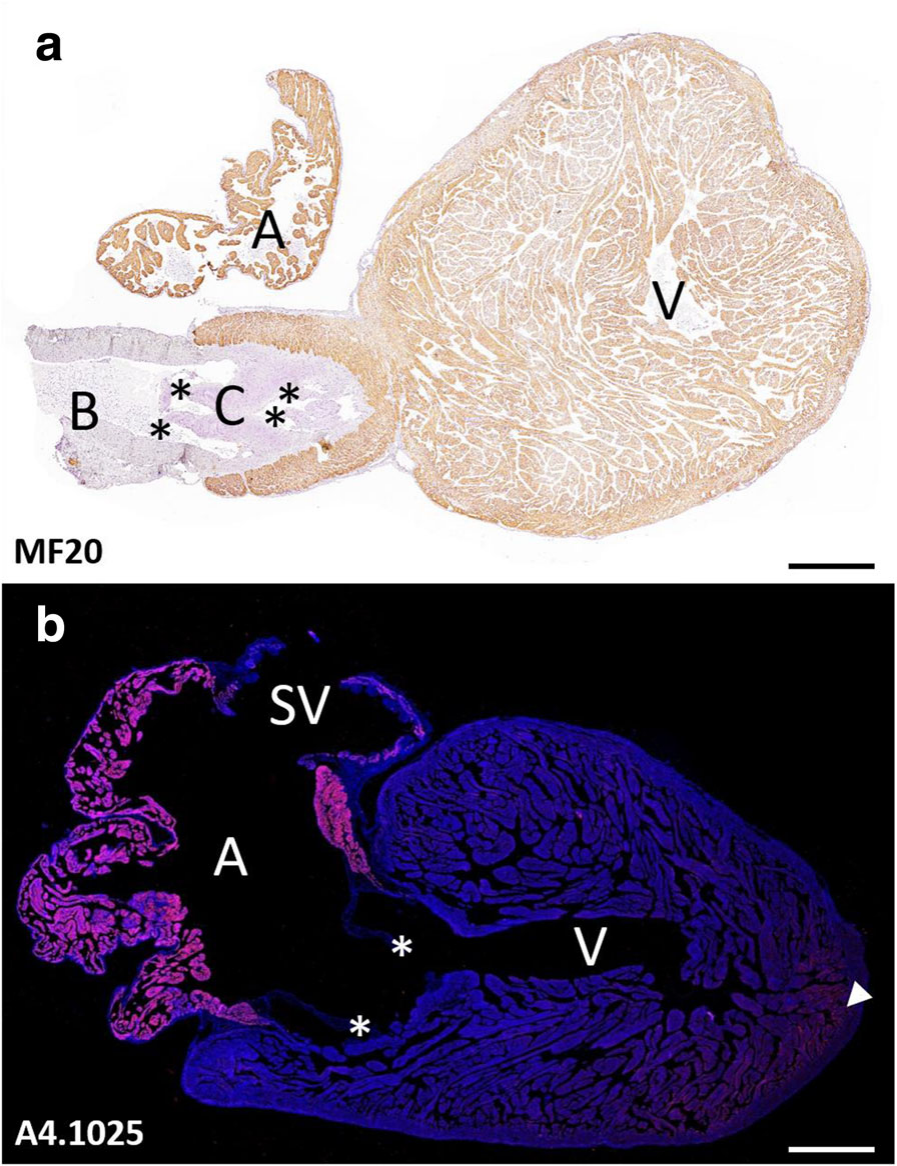
Sagittal sections of an adult heart from *Scyliorhinus canicula* immunolabeled with MF20 (**a**) and A4.1025 (**b**). While MF20 (brown) marks atrial (A), conal (C) and ventricular (V) myocardium, A4.1025 (pink) marks only the myocardium of the sinus venosus (SV), atrium (A) and the apex of the ventricle (arrowhead). B, bulbus arteriosus. Asterisks: cardiac valves. Scale bars: 1 mm

The heart of the dogfish is composed of five segments contained in the pericardial cavity, namely, the myocardial sinus venosus, atrium, ventricle and conus arteriosus and the non-myocardial bulbus arteriosus (Fig. 1). We will refer to the inflow segments when describing the sinus venosus plus atrium, and to the outflow segments when describing the ventricle plus the conus arteriosus. Immunohistochemical staining with the antibody MF20 revealed a homogeneous signal in all myocardial segments (Fig. 1a). However, immunohistochemistry in consecutive sections with the antibody A4.1025 showed signals restricted to the myocardium of the inflow segments (Fig. 1b). A less intense mark was detected in the ventricular apex.

Both MF20 and A4.1025 signals were specifically restricted to myocardial tissue. This is evident when comparing the strong labelling of the atrial wall with the absence of labelling of the conal and atrioventricular valves and the wall of the bulbus arteriosus (Fig. 1). In addition, the inflow segment restricted A4.1025 staining was evident when observing the limit between the atrial and ventricular walls.

**Fig. 2:**
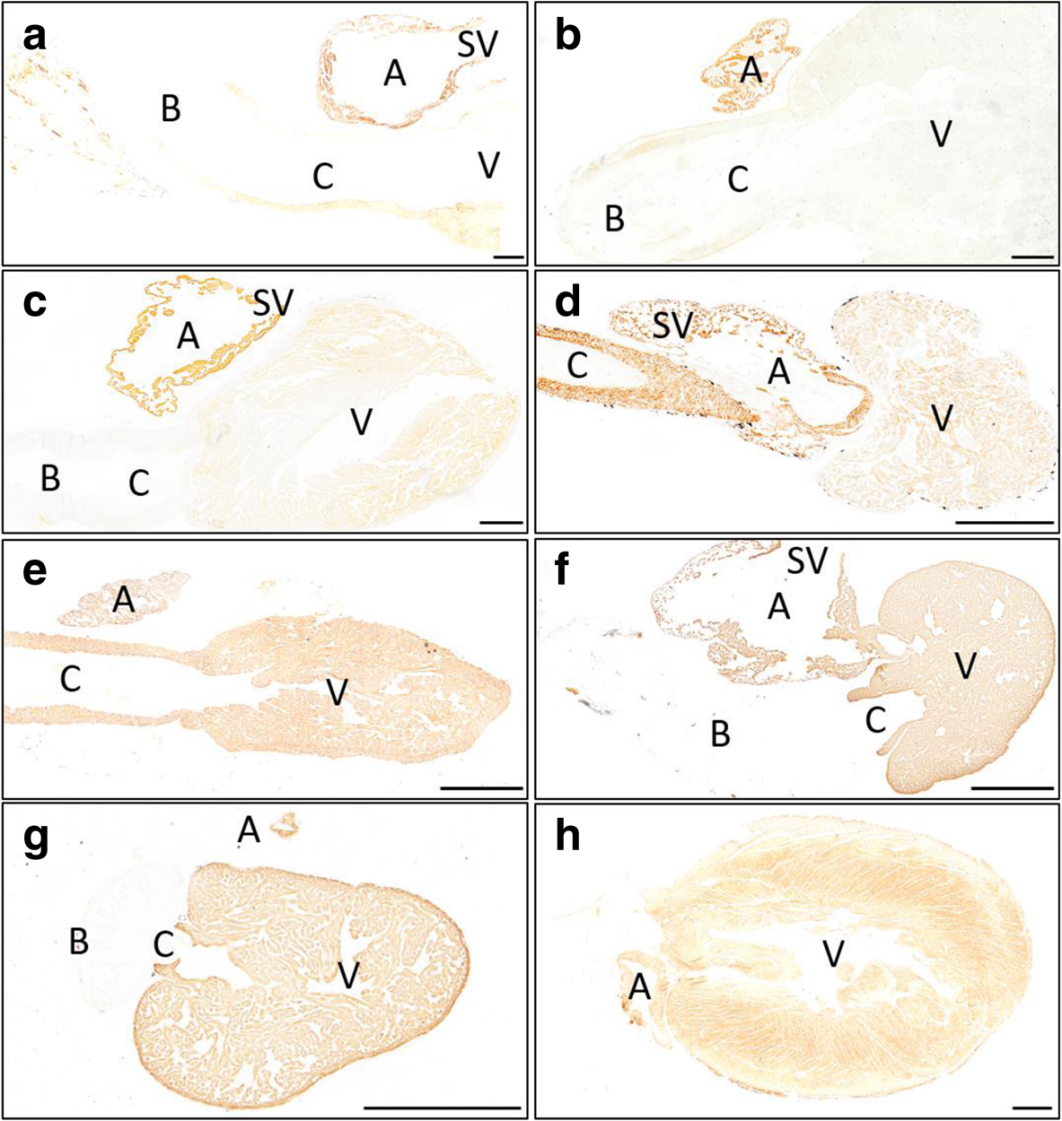
Sagittal sections of adult hearts from *Centrophorus granulosus* (**a**), *Leucoraja circularis* (**b**), *Scyliorhinus canicula* (**c**), *Polypterus senegalus* (**d**), *Acipenser naccarii* (**e**), *Osteoglossum bicirrhossum* (**f**), *Danio rerio* (**g**) and *Mesocricetus auratus* (**h**) immunolabeled with A4.1025. The signal is restricted to the atrial (A) and sinusal (SV) myocardium in chondrichthyans (**a**, **b**, **c**), to the sinusal (SV), atrial (A) and conal (C) myocardium in polypteriforms (**d**) and is present in all the myocardial segments of acipenseriforms (**e**), teleosts (**f**, **g**) and mammals (**h**). B, bulbus arteriosus; V, ventricle. Scale bars: 1 mm

To find out whether the distinct A4.1025 immunohistochemical pattern detected in the dogfish occurs in other vertebrates, we examined hearts from elasmobranchs (gulper shark and sand ray), actinopterygians (gray bichir, Adriatic sturgeon, silver arowana, zebrafish) and mammals (Syrian hamster). In all these species, MF20 staining was strong and homogeneous in the wall of all myocardial segments (not shown). Immunohistochemistry with A4.1025 in the hearts from sturgeons, arowanas, zebrafish and hamsters also revealed a strong and homogeneous signal in the whole myocardium (Fig. 2). However, the hearts of the gulper shark (Fig. 2a), sand ray (Fig. 2b), dogfish (Fig. 2c) and gray bichir (Fig. 2d) showed heterogeneous immunoreactivity against A4.1025 antibodies. In general, the staining pattern in the heart of the gulper shark (Fig. 2a) and sand ray (Fig. 2b) was similar to that detected in the dogfish, with intense signals in the inflow region and low or absent signals in the ventricle and conus (Fig. 2c). The pattern of A4.1025 staining in the heart of the gray bichir was different to that found in the other species examined. In this species, A4.1025 signal was strong in the sinus venosus, atrium and conus arteriosus, but faint in the ventricle (Fig. 2d).

### Slot and western blotting

**Fig. 3:**
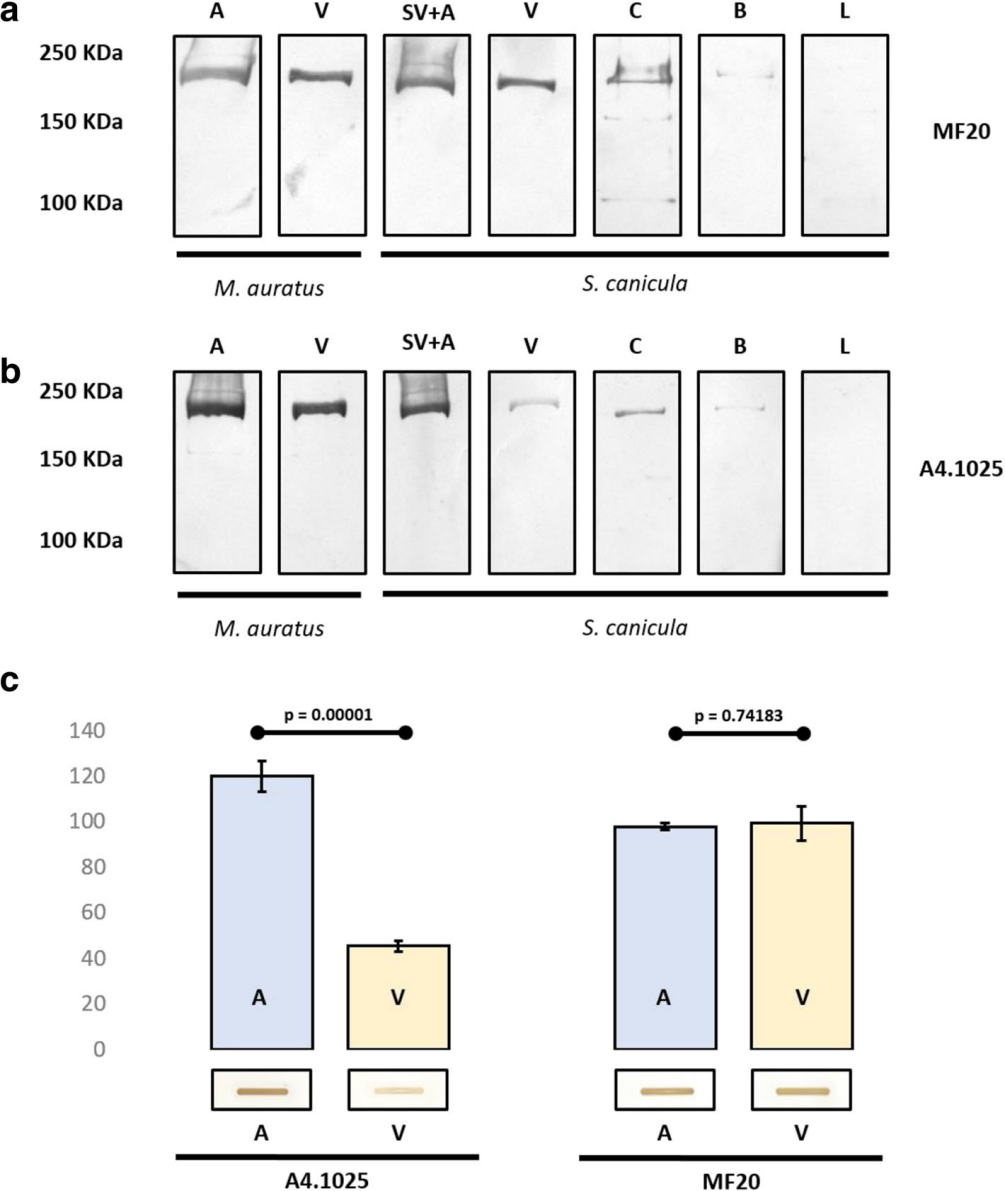
Myosin heavy chain immunochemical analysis of the atrium (A), and ventricle (V) of *Mesocricetus auratus* (**a**, **b**) and inflow tract (SV+A), ventricle (V), conus arteriosus (C), bulbus arteriosus (B) and liver (L) of *Scyliorhinus canicula* (**a**, **b**, **c**) using western blot (**a**, **b**) and slot blot (**c**) techniques. MF20 bands show a similar intensity in all the cardiac segments in *M. auratus* and *S. canicula* (**a**). A4.1025 bands are less intense in the ventricle and the conus arteriosus in the inflow tract of *S. canicula* (**b**). The slot blot colorimetric analysis (**c**) shows that the difference in intensity between the A4.1025 atrial and ventricular signals of *S. canicula* (*n*=5) is statistically significant (*p*=0.00001)

The fig. 3 shows western blots of MyHC extractions from different cardiac regions of hamsters and dogfish. In both species, a clear single band with a molecular weight between 200 kDa and 250 kDa was detected with both antibodies for all the cardiac segments, except for the bulbus arteriosus, which displayed a faint signal. In extracts from hamster hearts, the four bands obtained using both antibodies showed a similar strong intensity (Fig. 3a, b). In extracts from the dogfish hearts, the bands revealed with the MF20 antibody (Fig. 3a) corresponding to the atrium, ventricle and conus arteriosus showed a similar strong intensity. By contrast, those revealed with the A4.1025 antibody (Fig. 3b) corresponding to ventricle and conus arteriosus were markedly less intense.

To quantify the intensity of the signals detected from the protein extractions of the dogfish, we performed colorimetric analyses in bands obtained by means of slot blot assays (Fig. 3c). For these blots, we used a constant amount of MyHC proteins from the same extractions as in western blots. Atrial A4.1025 signals were almost three-fold more intense than ventricular bands (*p*=0.00001), whereas the intensity of MF20 atrial and ventricular signals showed no statistical differences (*p*=0.74183).

### Proteomic analysis by nano HPLC-ESI-MS/MS

The bands at 200-250 kDa corresponding to the myocardial inflow and outflow segments of dogfish samples were excised separately and subjected to peptide sequence analysis by nano HPLC-ESI-MS/MS. The resulting sequences were identified by MASCOT searching in a *Callorrinchus milii* (ghost or elephant shark) and in a chordate databases. A total of 275 sequence matches were found in the chordate database, 256 (93.03%) of which corresponded to isomyosins. In the *C. milii* database a total of 129 sequence matches were found, all of them corresponding to isomyosins.

From the inflow segments sample, the sequence matches detected in the *C. milii* database corresponded to four myosin isoforms: two cardiac isoforms (MYH6, MYH7), a skeletal isoform (MYH2) and a non-muscle isoform (MYH10) (Table 1). In the chordate database, the sequence matches corresponded to eight myosin isoforms: three cardiac isoforms (MYH6, MYH7, MYSC), four skeletal isoforms (MYH1B, MYH2, MYH8, MYH13) and a non-muscle isoform (MYH9) of a single species (Table 1).

**Table 1:** Results from peptide sequence analysis by nano HPLC-ESI-MS/MS of the inflow and outflow segments of *S. canicula*

Sample	Species	Protein name	Accession no	Mass (Da)	Score	N° Sequences
*S. canicula* Inflow segments	*C. milii*	Myosin 6	V9K8H5_CALMI	179853	15683	33
	*C. milii*	Slow myosin heavy chain 2	V9KCZ9_CALMI	120896	12224	26
	*C. milii*	Myosin heavy chain 7, cardiac	V9K7A6_CALMI	223387	3702	15
	*C. milii*	Myosin 10-like	V9K7K0_CALMI	192725	63	3
	*M. auratus*	Myosin 6	MYH6_MESAU	224287	20282	32
	*H. sapiens*	Myosin 7	MYH7_HUMAN	223757	15031	29
	*M. auratus*	Myosin 7	MYH7_MESAU	223646	14126	27
	*B. taurus*	Myosin 2	MYH2_BOVIN	224092	13777	22
	*M. musculus*	Myosin 8	MYH8_MOUSE	223653	13353	21
	*G. gallus*	Myosin 1B	MYH1B_CHICK	223648	11536	23
	*H. sapiens*	Myosin 13	MYH13_HUMAN	224605	7118	18
	*G. gallus*	Myosin heavy chain, cardiac	MYSC_CHICK	128216	6403	14
	*O. cuniculus*	Myosin 7	MYH7_RABIT	85835	4908	12
	*C. lupus familiaris*	Myosin 9	MYH9_CANLF	227583	203	10
*S. canicula* Outflow segments	*C. milii*	Myosin 6	V9K8H5_CALMI	179853	2609	20
	*C. milii*	Slow myosin heavy chain 2	V9KCZ9_CALMI	120896	2516	15
	*C. milii*	Myosin 7B-like	V9KBQ3_CALMI	131521	1196	5
	*C. milii*	Myosin, heavy chain 7, cardiac	V9K7A6_CALMI	223387	1102	8
	*C. milii*	Myosin 7-like	V9K9K7_CALMI	149632	529	2
	*C. milii*	Myosin 10-like	V9K7K0_CALMI	192725	52	2
	*M. musculus*	Myosin 8	MYH8_MOUSE	223653	615	9
	*M. musculus*	Myosin 7B	MYH7B_MOUSE	222501	512	6
	*M. auratus*	Myosin 6	MYH6_MESAU	224287	434	10
	*B. taurus*	Myosin 2	MYH2_BOVIN	224092	410	9
	*C. lupus familiaris*	Myosin 4	MYH4_CANLF	223776	393	9
	*O. cuniculus*	MyHC Embryonic smooth muscle	MYSU_RABIT	58084	28	1

Results from peptide sequence analysis by nano HPLC-ESI-MS/MS of the inflow and outflow segments of *S. canicula* (*n*=3) compared with a *C. milii* and a Chordate database. Samples from both inflow and outflow segments contained peptides corresponding to MYH2, MYH6, MYH7, and MYH10 whereas peptides corresponding to MYH7B were only detected in the outflow segments

From the outflow segments sample, the sequence matches detected in the *C. milii* database corresponded to six myosin isoforms: four cardiac isoforms (MYH6, MYH7, MYH7-like, MYH7B), a skeletal isoform (MYH2) and a non-muscle isoform (MYH10) (Table 1). In the chordate database, the sequence matches corresponded to six myosin isoforms: two cardiac isoforms (MYH6, MYH7B) of two species and four skeletal isoforms (MYH2, MYH4, MYH8; MYHemb) of four species (Table 1). All individual ions scores were >20 to indicate identity or extensive homology (*p*<0.01).

### Myosin heavy chain isoforms quantification by ESI-Quadrupole-Orbitrap

The bands at 200-250 kDa corresponding to the myocardial inflow and outflow segments of two additional dogfish samples were excised separately and subjected to peptide quantification by ESI-Quadrupole-Orbitrap. The resulting sequences were identified by SEQUEST searching in the same *C. milii* database.

The sequence matches detected in the *C. milii* database corresponded to the same isomyosins detected by nano HPLC-ESI-MS/MS (Table 2; MYH2, MYH6, MYH7 for the inflow segments and MYH2, MYH6, MYH7, MYH7B for the outflow segments). Traces of MYH9, MYH10 and MYH11 were additionally found in both segments, what can be explained by the higher sensitivity of this technique. All individual ions scores were >20 to indicate identity or extensive homology (*p*<0.01).

**Table 2:** Results from peptide sequence quantification by ESI-Quadrupole-Orbitrap of the inflow and outflow segments of *S. canicula*.

Protein name	Abundance average *(Inflow)*	Abundance average *(Outflow)*	% Isoform *(Inflow)*	% Isoform *(Outflow)*
Slow myosin heavy chain 2	169549940.70	93548252.81	34.3%	8.3%
Myosin-6	211090171.21	229440263.67	42.7%	20.4%
Myosin, heavy chain 7, cardiac muscle	108968584.27	511231134.91	22.0%	45.3%
Myosin-7B-like	195823.66	271036094.03	0.0%	24.0%
Myosin-10-like	2071101.07	9660654.80	0.4%	0.9%
Myosin-9-like	877770.49	2696819.69	0.2%	0.2%
Myosin-11	1731453.90	9714374.82	0.4%	0.9%

Results from Myosin heavy chain isoform average abundance (arbitrary units) and its percentage by ESI-Quadrupole-Orbitrap of the inflow and outflow segments of *S. canicula* (*n*=3) compared with a *C. milii* database. Both samples contain four sarcomeric isomyosins. The fast-twitch MYH2 and MYH6 are more abundant in the inflow segments whereas the slow-twitch MYH7 and MYH7B are predominant in the outflow segments. A small proportion (1-2%) of the non-sarcomeric isoforms MYH9, MYH10, MYH11 was found in both segments

The table 2 shows the average total and percental abundance of each MyHC isoform in the inflow and outflow segments. In the inflow segments MYH6 (42.7%) and MYH2 (34.3%) were the major isoforms, followed by MYH7 (22.0%), whereas MYH7B was almost absent. The non-sarcomeric isoforms MYH9, MYH10 and MYH11 represented less than 1% of the total myosins. In the outflow segments MYH7 was the major isoform (45.3%), followed by MYH7B (24.0%), MYH6 (20.4%) and MYH2 (8.3%). Non-sarcomeric isoforms (MY9, MYH10 and MYH11) represented around 2% of the total myosins.

### A4.1025 immunoprecipitation and isoform antibody affinity analysis

One of the myosin extractions of the dogfish outflow used for ESI-Quadrupole-Orbitrap was immunoprecipitated using an affinity column coated with A4.1025. The bands at 200-250 kDa corresponding to the initial extraction and the same extraction after column elution were excised separately and subjected to peptide quantification by ESI-Quadrupole-Orbitrap. The resulting sequence were identified by SEQUEST searching in the *C. milii* database.

The cardiac MyHC sequence matches detected in the *C. milii* database from both bands corresponded to the same isomyosins detected by nano HPLC-ESI-MS/MS and ESI-Quadrupole-Orbitrap in previous experiments (compare Table 1 with Tables 2 and 3). The table 3 shows the total and percentual abundance of each cardiac MyHC isoform in both the initial extraction and the eluate. The isomyosin abundances of the initial extraction showed similar values to those obtained in the previous experiments (MYH7=49.1%; MYH7B=28.3%; MYH6=15.4%; MYH2=7.2%). However, the isomyosin abundances after A4.1025 column elution changed significantly. While MYH7 showed a relative abundance similar to that in the original extract (46.7%), MYH2 and MYH6 were overrepresented (15.3% and 31.4%, respectively) and MYH7B was reduced almost four folds (6.5%). The relative affinity of the A4.1025 for the different cardiac MYH isoforms in the dogfish outflow segments was estimated by dividing the relative amount of each isoform in the eluate by the relative amount in the initial extraction. MYH2 and MYH6 showed more than double affinity for A4.1025 than MYH7, and almost 10 times more affinity than MYH7B.

**Table 3:** Abundance and relative proportion of sarcomeric isomyosins of the outflow segments before and after A4.1025 immunoprecipitation and A4.1025 relative affinity of the four isoforms

Protein name	Abundance *(Extract)*	Abundance *(Eluted extract)*	% Isoform *(Extract)*	% Isoform *(Eluted extract)*	A4.1025 Relative affinity
Slow myosin heavy chain 2	90803168.43	54922737.11	7.2%	15.4%	2.14
Myosin-6	195381851.59	112643694.43	15.3%	31.4%	2.04
Myosin, heavy chain 7, cardiac muscle	623452935.20	167566431.54	49.1%	46.7%	0.95
Myosin-7B-like	359536869.87	23369498.76	28.3%	6.5%	0.23

Results from Myosin heavy chain isoform abundance (arbitrary units) by ESI-Quadrupole-Orbitrap of the outflow segments of *S. canicula* before and after immunoprecipitation with A4.1025. The A4.1025 relative affinity is the quotient between the concentration of isomyosins before and after immunoprecipitation. MYH2 and MYH6 showed more than double affinity for A4.1025 than for MYH7, and almost 10 times more affinity than for MYH7B

## Discussion

In most vertebrates studied until now, i.e. mammals, birds and teleosts, two MyHC isoforms, MYH6 and MYH7, are expressed in the adult heart. Orthologs of MYH7 are predominantly expressed in the ventricle, whereas orthologs of MYH6 are predominantly expressed in the atrium and, in fish species, also in the sinus venosus. This data was obtained by means of *in situ* hybridization [4, 6, 49, 59, 60] and electrophoretic separation [34, 39]. One exception is *Xenopus*, in which, as Garriock et al. [17] described after sequence comparison and *in situ* hybridization, an ortholog of MYH15 is the main ventricular MyHC isoform. MYH15 has become a pseudogene in mammals [17]. However, the isomyosin distribution in the heart of chondrichthyans, the earliest phylogenetical group of living gnatosthomes, remains unknown. Knowledge of MYH isoform expression in the chondrichthyan heart would inform about (1) what was the primitive condition of the trait in gnatosthomes, and (2) when the cardiac MYH distribution shared by most gnatosthomes did appear.

In an ongoing immunohistochemical study of the chondrichthyan heart using the dogfish as an animal model, we detected a striking pattern of immunohistochemical signals with the two anti-pan-MyHC antibodies MF20 and A4.1025. While MF20 signals were homogeneously distributed in the four myocardial segments of the chondrichthyan heart, namely, the sinus venosus, atrium, ventricle, and conus arteriosus, A4.1025 signals were strong in the sinus venosus and atrium, but faint in the conus arteriosus and the ventricle (Fig. 1). This segment-specific immunohistochemical pattern differs from the homogeneous pattern detected with A4.1025 in all the vertebrate species examined, i.e. hamsters (present results), mice [29, 52], *Xenopus* [1, 44, 48] and zebrafish [22, 24, 44]. This data already suggests that the cardiac segments of the dogfish heart express distinct MyHC isoforms that show differential affinity for MF20 and A4.1025 antibodies.

In order to corroborate our immunohistochemical results, we performed western blot analyses using MF20 and A4.1025 antibodies. Single 220 kDa bands, the approximate molecular weight of MyHC [47], were obtained after applying a myosin-specific extraction method in dogfish and hamster hearts. This confirms the specificity of both antibodies for MyHC in both species. In addition, the different intensity between the A4.1025 bands corresponding to the inflow and outflow myocardial segments of the dogfish heart strengthens the immunohistochemical findings. Western blot results were corroborated by colorimetric quantification of bands obtained in slot blot analyses. In the dogfish, the intensity of A4.1025 signals in the outflow segments was almost three**-**fold significantly lower than that in the inflow segments, whereas the intensity of MF20 signals did not significantly differ between blots.

The wall of the bulbus arteriosus does not contain myocardium. It is mainly composed of smooth muscle cells, elastin and collagen [13, 30]. However, our western blot analyses of bulbus extracts showed a narrow band using MF20 or A4.1025 antibodies (Fig. 3). In this regard, we cannot exclude that myocardial tissue of the distal portion of the conus arteriosus was removed together with the bulbus during dissection for protein extraction.

MF20 and A4.1025 antibodies recognize different epitopes of the MyHC protein. MF20 is specific for the tail region [2], whereas the epitope recognized by A4.1025 is in the head region [7, 10]. Thus, our immunohistochemical, western blot and slot blot results strongly suggest that in contrast to the mammalian heart, in the dogfish heart there is at least one MyHC isoform with a different affinity for MF20 and A4.1025 antibodies. In addition, it can be deduced that this or these isoforms are differentially expressed in the inflow versus the outflow myocardial segments in this species.

To test our hypothesis and identify the MyHC isoform/s differentially expressed in the dogfish cardiac segments, we performed HPLC-ESI-MS/MS analyses in the samples of the inflow and outflow segments. The genomic DNA of the dogfish has not been fully sequenced, and there is no record of the peptide sequence of the MyHC isoforms in this species. Although the whole genome of three additional elasmobranch species (*Chiloscyllium punctatum*, *Scyliorhinus torazame* and *Rhincodon typus*) has been recently sequenced [19], these sequences are unassembled, and hence they are not accessible to the MS search engine. Therefore, we compared the trypsinised peptides of the inflow and the outflow tract myocardium of the dogfish with protein sequences obtained from the genomic database of the elephant shark (*Callorhinchus milii*) provided by Venkatesh et al. [54]. *C. milii*, which belongs to the holocephalans, the sister group of the elasmobranchs, is the only chondrichthyan with the MYH protein sequences included in the UniProtKB/Swiss-Prot database. The MS search engine used in this study (MASCOT and SEQUEST) acquires its maximal efficiency when the highly reliable UniProtKB/Swiss-Prot database is used for comparisons. Samples from both the inflow and outflow segments of the dogfish contained peptides corresponding to MYH10, MYH2, MYH6 and MYH7, whereas peptides corresponding to MYH7B were only detected in the outflow segments (Table 1). In order to confirm these results, we used a chordate peptide database. As it might be expected taking into account the high number of entries in the chordate database and the high level of conservation of MyHC isoforms, several additional sequences matched with peptides in our samples (MYH1B, MYH4, MYH8, MYH9, MYH13, MYHemb and MYSC). Despite this, MYH7B was again found to match with peptides from the outflow segments but not with those from the inflow segments (Table 1).

In order to confirm the distribution of MyHC isoforms revealed by HPLC-ESI-MS/MS and to quantify the relative abundance of these isoforms, we performed ESI-Quadrupole-Orbitrap assays in additional dogfish samples. The results confirmed the data obtained by the previous experiments. The dogfish cardiac outflow segments contain four main sarcomeric isomyosins: MYH2, MYH6, MYH7 and MYH7B, whereas the inflow segments contain only MYH2, MYH6 and MYH7. In addition, the relative abundance of these isoforms is segment-specific. The fast-twitch MYH2 and MYH6 account for more than 75% of the total MyHC isoforms in the inflow segments, where MYH7 (22%) is the only slow-twitch isoform. By contrast, the two slow-twitch isoforms MYH7 and MYH7B are predominant in the outflow segments (70%), whereas MYH2 and MYH6 account for less than 30% of total isomyosins in these segments.

According to the contraction speed, there are two types of skeletal isomyosins: slow and fast-twitch isoforms. In all the vertebrates studied until now, the fast-twitch MyHC that characterizes the inflow segments is MYH6, whereas the slow-twitch MYH7 predominates in the ventricle [15]. Our results show that the myocardium of chondrichthyans contains two additional MyHC isoforms, which are not expressed in the heart of other vertebrates, MYH2 (fast-twitch), abundant in the inflow segments, and MYH7B (slow-twitch), concentrated in the outflow segments. Nevertheless, the myocardium of chondrichthyans shows the same segment-specific composition of isomyosins as other vertebrates with respect to their contraction speed: the inflow segments contains predominantly fast-twitch isomyosins (MYH2 and MYH6; 77%), while the outflow segments contains predominantly slow-twitch isomyosins (MYH7 and MYH7B; 70%). Thus, this segment-specific distribution of fast *vs.* slow MyHC isoforms can be considered a shared trait of Gnathostomes. New studies in cyclostomes may help to elucidate whether this trait is a synapomorphy or a symplesiomorphy for Gnathostomes. However, the composition of specific cardiac isomyosins has changed during the evolution of the group, reducing its variability in derived taxa.

MYH2 is a fast-twitch skeletal muscle MyHC ([25]), which has not been detected in the myocardium of any adult vertebrate studied until now. Our results point to two possible evolutionary scenarios. In early gnathostomes, MYH2 may have been a cardiac myosin isoform that lost its function in cardiac contraction throughout evolution. Given that MYH2 is absent in the heart of both mammals and teleosts, loss of cardiac expression of this isomyosin might have taken place before the origin of Osteichthyes. Alternatively, MYH2 cardiac expression may have been absent in early gnathostomes, being an independent acquisition in chondrichthyans. MYH7B (firstly known as MYH14) is a poorly understood slow tonic MyHC isoform which, together with MYH15 and MYH16 is considered an ancient MyHC isoform that could have given rise to the current main cardiac isomyosins, MYH6 and MYH7, after a duplication process [12, 53]. Although MYH7B transcripts have been detected in the heart of multiple vertebrate species, including human, mouse [56], rat [40], chick, *Xenopus* [56] and zebrafish [28], the cardiac expression of the protein has been the subject of debate. Warkman et al. [57] self-dismissed a previous positive result in the adult human heart due to a failure in their early experimental design. In addition, it was not clear if an ortholog of MYH7B (ssMHC) is really expressed in the cardiac conduction system of the chick [32], because later experiments were not able to reproduce this result [56]. In mammals, MYH7B protein has only been found in the extraocular musculature of humans and rats [40], and in the *soleus* muscle of mice [3]. Absence of MYH7B expression in the cardiac muscle relies on the presence of a premature termination codon in exon 7 of the gene. The activation of a splicing machinery in skeletal muscle that excludes this exon allows MYH7B translation in this tissue [3]. Therefore, our results clearly show for the first time the expression of MYH2 and MYH7B protein in the adult myocardium of an extant vertebrate, namely, the lesser spotted dogfish.

Similar to *myh6* and *myh7*, *myh7b* DNA sequence includes an intronic miRNA (MyomiR) named miR-499 [53], which is present in the *myh7b* gene of most vertebrate species [5]. The importance of miR-499 relies on its function as a post-transcriptional regulation element of *myh7b* [3]. It has been also proposed that miR-499 is able to interact with *Sox6* to regulate the pathway responsible for the differentiation of the slow and the fast musculature [55]. We propose that in teleosts, birds and mammals MYH6 and MYH7 became mainly restricted to the inflow and outflow segments respectively, whereas MYH2 and MYH7B disappeared from the heart. The mRNA of MYH7B probably acquired a regulatory role in cardiac and extracardiac myogenesis through its intronic miRNA, miR-499 [5, 28, 35, 55].

Using a combination of Orbitrap-Quadrupole and A4.1025 immunoprecipitation methodologies, we were able to calculate the relative affinity of the MyHC isoforms of the dogfish heart for the A4.1025 antibody. The slow-twitch MyHC isoforms showed a reduced affinity compared to the fast-twitch isoforms, being MYH7B particularly unable to bind the antibody (Table 3). These data allow interpretation of the A4.1025 immunohistochemical results in terms of MyHC isoform content. The strikingly low A4.1025 signals in the cardiac outflow segments of the dogfish (Fig. 1) is most probably due to three factors: 1) the high proportion of slow-twitch MyHC isoforms in these segments; 2) the low affinity of these isoforms (particularly MYH7B) for A4.1025; 3) the low concentration of total MyHC in the outflow compared with the inflow segments (see [16]).

**Fig. 4:**
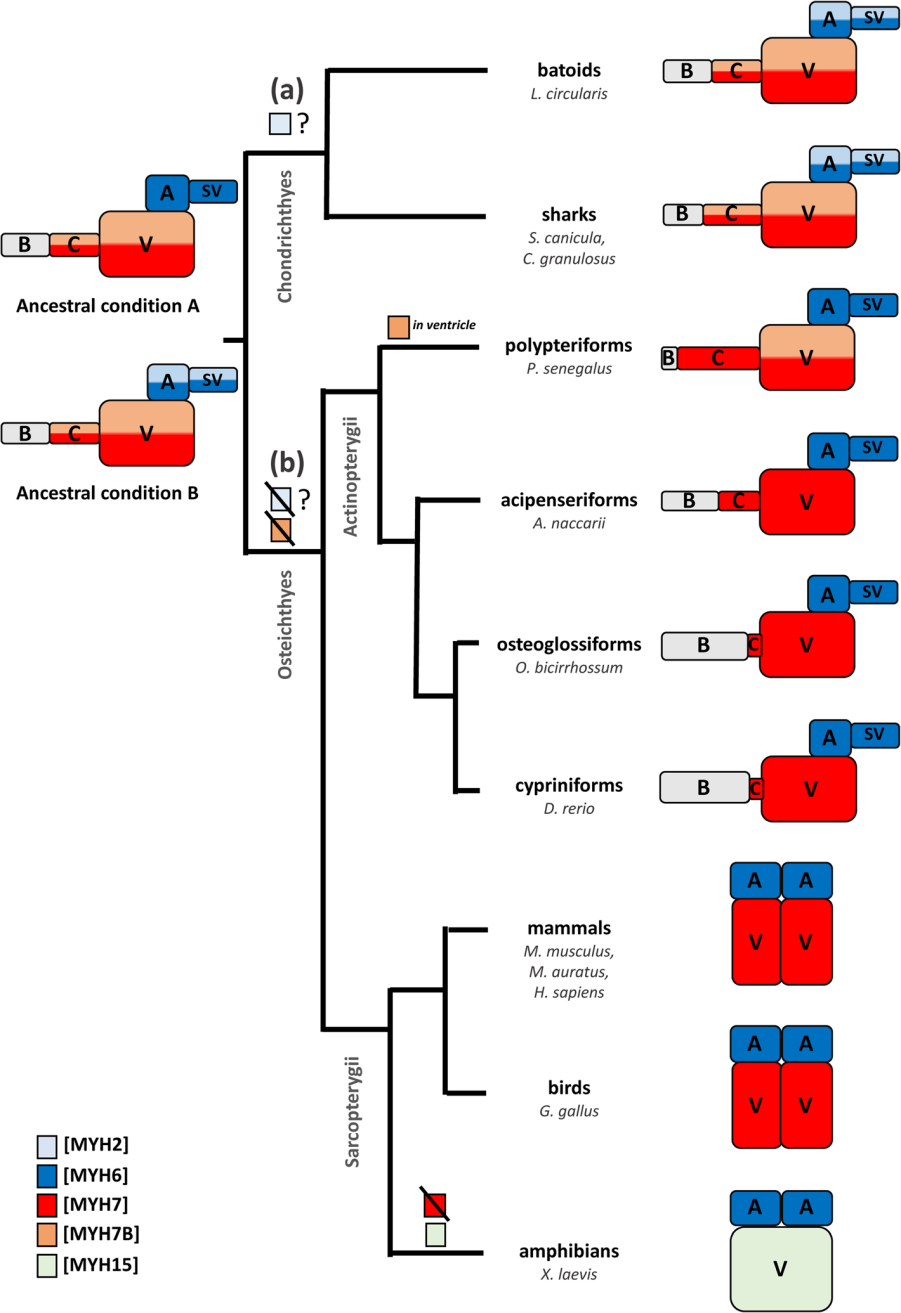
Phylogenetic tree of gnathostome taxa (based on [8]) and the distribution of the five cardiac MyHC isoforms according to the two hypothesis raised in the present study. The colored boxes indicate the five MyHC isoforms found in the vertebrate heart. The colors in the diagrams indicate predominant isoforms in each cardiac segment of the nine taxa in which the trait is known, either from the present results or from previous studies. In the evolutionary scenario (**a**), the expression of three cardiac isomyosins (MYH6, MYH7 and MYH7B) is referred as the ancestral condition A. MYH2 expression in the inflow segments would be an acquisition of Chondrichthyes. In the alternative evolutionary scenario (**b**), the ancestral condition B is characterized by the expression of four cardiac isomyosin (MYH2, MYH6, MYH7 and MYH7B). In both evolutionary scenarios, MYH6 and MYH7 became the only cardiac isomyosins in osteichthyes, except for polypteriforms and amphibians. The former reacquired MYH7B expression in the ventricle, whereas the latter changed MYH7 by MYH15 in this segment. To elaborate this phylogenetic tree, data for sharks come from proteomic analysis (ESI-Quadrupole-Orbitrap, present results) whereas data for batoids, polypteriforms and osteoglossiforms come from immunohistochemistry (present results), for cypriniforms, amphibians and birds using *in situ* hybridization [4, 6, 17, 49, 59, 60] and for mammals come from of electrophoretic separation [34, 39]. See the text for details. A, atrium; B, bulbus arteriosus; C, conus arteriosus; SV, sinus venosus; V, ventricle

If we assume that the A4.1025 antibody has similar affinities for MyHC isoforms of different gnathostomes, given the high level of conservation of the ATP-binding site in the MyHC protein sequences among vertebrates, our initial immunohistochemical study of different vertebrate species (Fig. 2) can be reinterpreted in terms of specific isomyosin abundance. The sand ray and the gulper shark showed the same A4.1025 immunohistochemical pattern as the dogfish, suggesting a distinctly shared MyHC distribution in the heart of chondrichthyans (MYH2 and MYH6 in the inflow and MYH7 and MYH7B in the outflow segments). The silver arowana, the sturgeon, the zebrafish and the hamster showed the expected A4.1025 homogeneous immunohistochemical pattern in all the myocardial segments, suggesting that the conventional MyHC distribution (MYH6 in the inflow and MYH7 in the outflow segments) appeared at the base of Osteichthyes. By contrast, the gray bichir, a representative of polypteriforms, the earliest diverged group of extant actinopterygians [27, 51], showed a different A4.1025 immunohistochemical pattern of staining compared to all the other species studied. A4.1025 signals were intense in all the myocardial segments except for the ventricle. This can be interpreted as a relatively high abundance of slow-twitch isomyosins, particularly MYH7B, only in the ventricle but not in the conus arteriosus. From the evolutionary viewpoint, this finding implies a reacquisition of MYH7B expression in the heart of polypteriforms (Fig. 4). In addition, it will be interesting to investigate possible differences in the type of myocardial contraction between bichirs and other actinopterygians. Further studies are required to test the hypothesis depicted in fig. 4, by analyzing the MyHC isoform distribution in the heart of additional vertebrate species, particularly in sarcopterygians and other early diverged actinopterygians (as lepisosteiforms) would be of high interest.

In gnathostomes there are two basic cardiac anatomical designs, primitive and modern with respect to the type of cardiac contraction [18, 30]. In mammals, birds and most teleosts, which show the modern design, the main contractile segments are the atrium and the ventricle, whereas in chondrichthyans and early actinopterygians, which show the primitive design, the conus arteriosus is interposed between the ventricle and the bulbus arteriosus. While atrium and ventricle work as synchronous, fast-contracting segments, the conus arteriosus is a slow-contracting segment with peristaltoid contraction. It has been hypothesized that the conus arteriosus serves as an accessory pumping chamber [50], as an elastic reservoir to minimize pressure fluctuations [9, 26], and to allow a correct function of the conal valvular apparatus [41] in elasmobranchs. It has been also proposed that the myocardium of the conus arteriosus of teleosts plays an active function in the performance of the outflow tract valves [42]. Birds and mammals also show the primitive cardiac anatomical design at early embryonic stages [11, 16, 37]. In these embryos, MYH isoform coexpression in the ventricle and the conus arteriosus, including MYH7B at least in mammals [56], allows a slow conduction velocity and a peristaltoid contraction [46]. After cardiac septation, a shift to segment-specific MYH single isoform expression allows development of the fast conduction velocity and synchronous contraction of atrium and ventricle, necessary for the adult type of one-way valves that start to develop at the end of septation (Moorman & Lammers, 1994). According to our results, MYH7B is expressed in the ventricle and/or the conus arteriosus of Chondrichthyes and Polypteriforms, which are fish with a primitive cardiac anatomical design, i.e. with a well-developed conus arteriosus. It can then be hypothesized that the slow tonic MYH7B is required for the peristaltic contraction of the conus arteriosus in fish with a primitive cardiac anatomical design. MYH7B peptides would have disappeared from the adult heart once the modern design, including a synchronous and fast contraction, has developed in tetrapods and teleosts. The MyHC isoform expression pattern in polypteriforms and acipenseriforms, both with a long conus but a different MYH7B cardiac content compared to chondrichthyans, may represent transitional conditions between the primitive and the modern cardiac anatomical design. In this context, it would be interesting to further investigate the anatomy, type of contraction and precise MYH isoform composition in these two actinopterygian groups.

## Conclusion

Similar to the other gnathostomes, the cardiac inflow segments of the dogfish show predominance of fast-twitch MyHC isoforms whereas the outflow segments show predominance of slow-twitch isoforms. Therefore, the segment-specific distribution of fast- *vs.*slow-twitch isomyosins in the heart can be considered a synapomorphy or a symplesiomorphy for gnathostomes, pending the analysis of cyclostomes.

Besides the two conventional cardiac MyHC isoforms (MYH6 and MYH7), the myocardium of the dogfish contains two additional isomyosins (MYH2 and MYH7B), not expressed in the heart of other vertebrates. These two isoforms may have lost their function in cardiac contraction during the evolution of gnathostomes, the latter acquiring a regulatory role in cardiac and extracardiac myogenesis through its intronic miRNA. Our results suggest that this change in the distribution of cardiac MyHC isoforms occurred before the origin of Osteichthyes, and that polypteriforms reacquired ventricular MYH7B expression.

We raise the hypothesis that the slow tonic MYH7B may facilitate the peristaltic contraction of the conus arteriosus of fish with a primitive cardiac anatomical design and of the vertebrate embryo.

## Methods

### Animals

The sample examined consisted of 31 adult hearts of the following eight gnathostome species: *Leucoraja circularis* (sandy ray; *n*=2) and *Centrophorus granulosus* (gulper shark; *n*=2) both collected by scientific vessels of the Spanish Institute of Oceanography fleet; *Scyliorhinus canicula* (lesser spotted dogfish; *n*=15), *Polypterus senegalus* (gray bichir, *n*=2), *Acipenser naccarii* (Adriatic sturgeon, *n*=2), *Osteoglossum bicirrhossum* (silver arowana, *n*=2), and *Danio rerio* (zebrafish, *n*=2) from local suppliers, and *Mesocricetus auratus* (golden hamster, *n*=4) from an outbred strain commercialized by *Janvier* (RjHan: AURA; France).

The animals were handled in accordance with the European and Spanish guidelines for animal welfare. The animals processed in the laboratory were killed by anaesthetic overdose with 0.04% MS-222 (Sigma-Aldrich Chemical Co., Poole, UK) in freshwater for the actinopterygians, or by CO_2_ inhalation for hamsters. The ventral region of the pericardial cavity was exposed by means of a longitudinal incision along the anterior, midventral line of the animal, and the heart was removed and rinsed in elasmobranch buffer (16.38 g L^-1^ NaCl, 0.89 g L^-1^ KCl, 1.11 g L^-1^ CaCl_2_, 0.38 g L^-1^ NaHCO_3_, 0.06 g L^-1^ NaHPO_4_, 21.6 g L^-1^ urea, pH 7.2) for the chondrichthyans or 0.1M phosphate buffer saline (PBS; pH 7.3) for the actinopterygians and mammals. For the proteomic analyses of samples from *S. canicula* and *M. auratus*, inflow and outflow segments were dissected through the atrioventricular and conoventricular limits or through the atrioventricular and ventriculoarterial limits, respectively.

### Immunohistochemical techniques

Five hearts from *S. canicula* and two from each of the other species used in this work were fixed in methanol/acetone/water (2:2:1) or in 4% paraformaldehyde diluted in elasmobranch buffer for chondrichthyans or in PBS for actinopterygians and mammals. Then, the hearts were dehydrated in a graded series of ethanol and embedded in Histosec paraffin (Merck KGaA; Darmstadt, Germany). Serial sagittal sections of hearts cut at 10 μm were dewaxed, hydrated and washed in Tris-PBS (TPBS, pH 7.8). For immunoperoxidase, endogenous peroxidase activity was quenched by incubation for 30 min with 3% hydrogen peroxide in TPBS. After washing with TPBS, non-specific binding sites were saturated for 30 min with 10% sheep serum, 1% bovine serum albumin (BSA) and 0.1% Triton X-100 in TPBS (SBT). The slides were then incubated overnight at 4°C in the primary antibody diluted in SBT. Negative control slides were incubated in SBT only. After incubation, the slides were washed in TPBS, incubated for 1.5 h at room temperature in biotin-conjugatedanti-mouse IgG (Sigma-Aldrich Chemical Co., Poole, UK) diluted 1:1000 in SBT, washed again and incubated for 1 h in ExtraAvidin®-peroxidase complex (Sigma-Aldrich Chemical Co., Poole, UK) diluted 1:250 in TPBS. Peroxidase activity was detected using Sigma Fast 3,3’-diaminobenzidine tablets (Sigma-Aldrich Chemical Co., Poole, UK) following the indications of the supplier. Some sections were counterstained with hematoxylin staining. The sections were observed with a Leica DMSL light microscope or with an Olympus VS120 virtual microscopy slide scanning system (Olympus, Tokyo, Japan) equipped with the VS-ASW software (Olympus, Tokyo, Japan) and viewed using the free of charge software OlyVIA (Informer Technologies, Inc., Walnut, CA, USA).

In selected sections, ExtraAvidin®-peroxidase and further incubations were replaced by ExtrAvidin-Cy3™ (Sigma-Aldrich Chemical Co., Poole, UK) incubation and counterstaining with 4’,6-diamidino-2-phenylindole dihydroclhoride (DAPI) (Sigma-Aldrich Chemical Co., Poole, UK). They were observed and photographed with an Olympus VS120 virtual microscopy slide scanning system (Olympus, Tokyo, Japan).

The primary antibodies were MF20 (DSHB Cat# MF-20, RRID: AB_2147781) and A4.1025 (DSHB Cat# A4.1025, RRID: AB_528356) both anti-MyHC monoclonal antibodies (Developmental Studies Hybridoma Bank, University of Iowa). They were used at dilutions of 1:20 and 1:200 of the respective supernatants.

### Western blot, slot blot and colorimetric analysis

For western and slot blot analyses, MyHC was extracted and quantified from tissue lysates following a specific protocol for MyHC [33]. Protein concentrations were evaluated with a NanoDrop ND-1000 spectrophotometer (Nanodrop technologies, Wilmintogton, DE, USA). A total of five adult hearts from *S. canicula* and two from *M. auratus* were used.

For western blot analysis, 1 μg of protein was diluted in 10 μL of sample buffer (Tris 0.312 M pH 6.8 with 5% SDS, 0.25% bromophenol blue, 50% glycerol and 2-mercaptoethanol 0.9 M), heated at 100°C for 5 min and resolved by SDS-PAGE in an 8% acrylamide gel. Electrophoresis was performed at 125 V for 2 h. Proteins were transferred onto nitrocellulose membranes using a Trans-Blot SD semi-dry blotting system (BioRad Laboratories, California, USA) run at 15V for 1.5 h. After washing with TPBS, non-specific binding sites were saturated for 30 min with 5% milk in TPBS. The membranes were then incubated overnight at 4°C in the primary antibody diluted in SBT. MF20 and A4.1025 were used at 1:1000 and 1:2000 dilution of the supernatants, respectively. After incubation, the membranes were washed in TPBS, incubated for 1.5 h at room temperature in biotin-conjugatedanti-mouse IgG (Sigma-Aldrich Chemical Co., Poole, UK) diluted 1:1000 in SBT, washed again and incubated for 1 h in avidin-peroxidase complex (Sigma-Aldrich Chemical Co., Poole, UK) diluted 1:250 in TPBS. Peroxidase activity was detected using the same protocol as for immunohistochemistry. The bands obtained were photographed with a Gel Doc™ XR+ System (BioRad Laboratories, California, USA) equipped with the Image Lab software (BioRad Laboratories, California, USA).

For slot blot analysis, nitrocellulose membranes were pre-soaked in PBS for five min. Fifteen μL of myosin extraction (3 μg/μL) were added to the blotter (Hoefer Scientific Instrument, San Francisco, USA) applying vacuum. The membranes were removed and washed in TPBS. Non-specific binding sites were saturated for 30 min with 5% milk in TPBS. The membranes were then incubated overnight at 4°C in the primary antibody diluted in SBT. MF20 and A4.1025 were used at 1:1000 and 1:2000 dilution of the supernatants, respectively. After incubation, the membranes were washed in TPBS, incubated for 1.5 h at room temperature in biotin-conjugatedanti-mouse IgG (Sigma-Aldrich Chemical Co., Poole, UK) diluted 1:1000 in BSA, washed again and incubated for 1 h in avidin-peroxidase complex (Sigma-Aldrich Chemical Co., Poole, UK) diluted 1:250 in TPBS. Peroxidase activity was detected using the same protocol as for immunohistochemistry. The membranes were photographed in black and white. The intensity of the signal in each band was measured using the software FIJI [43]. Average data from five specimens were obtained and statistical differences were tested using t-student test.

### Sample preparation for proteomic analysis

All proteomic analyses were performed at the Proteomics Core Facility of the University of Málaga. The bands identified on the western blot were isolated from the gel, destained, reduced with DTT, alkylated with iodoacetamide and digested in-gel with trypsin (Promega, Southampton, UK) automatically in a DigestPro MSI (INTAVIS Bioanalytical Instruments AG, Köln, Germany). Bands corresponding to extractions from atrial, sinusal, ventricular and conal myocardium were used.

### Nano HPLC-ESI-MS/MS analysis

For the analysis of the trypsinized peptides, liquid chromatography was performed using a Nano-HPLC Agilent 1200 comprising a 1200 Series G1379B Degasser, a 1200 Series G2226A Nano pump, 1200 Series G1330B Thermostatted controller, 1200 Series G1377A Micro well plate Autosampler, a 1200 Series G1316B column compartment, a 1200 Series 4208A Gameboy controller and a 1200 Series Solvent Tray (Agilent Technologies, Santa Clara, CA, USA).

Samples were concentrated and desalted in the trapping column C18, ZORBAX 300SB-C18 (Agilent Technologies, Germany), 5x0.3 mm, 5 μm i.d. using a gradient of 98% H_2_O: 2% acetonitrile (ACN) containing 0.1% formic acid (FA), with a flow rate of 20 μL/min for 6 min. The trapping column was connected in line with the analytical column ZORBAX 300SB-C18 (Agilent Technologies, Germany), 150x0.075mm, 3.5 μm i.d. Elution of the trapping column samples was performed using a gradient of two solvents: A (0.1% FA in H_2_O) and B (0.1% FA in 80% ACN and H_2_O), with a flow rate of 300 nL/min. The elution gradient scheme was: isocratic conditions of 98% A: 2% B for 6 min, lineal increase to 50% B in 120 min, lineal increase to 90% B in 2 min, isocratic conditions B 90% for 3 min and back to the initial conditions in 3 min. The ejection volume was 20 μl. Nano-HPLC was interfaced directly with a 3D high capacity ion trap mass spectrometer AmaZon speed ETD (Bruker Daltonics; Billerica, Massachusetts, USA) using CaptiveSpray nanoBooster (Bruker Daltonics; Billerica, Massachusetts, USA) and nano-ESI mode. Ion trap was turned for a target mass of 300-1500 m/z and a smart ICC target of 200.000.

The raw HPLC-MS/MS data obtained were processed using ProteinScape 3 (Brunker Daltonics; Billerica, Massachusetts, USA). For protein identification, MS/MS spectra were compared with UniProtKB/Swiss-Prot database (Release 2017_02, 15-Feb-17, number of entries 553655). Searches were done using Mascot v.3.1 (http://www.matrixscience.com; Matrix Science, London, U.K.). Search parameters were set as follows: carbamidomethyl cysteine as fixed modification and oxidized methionine as variable modification. Peptide mass tolerance was set at ± 0.6 Da in the precursor ions and ± 0.5 Da to the fragmented mases in the MS/MS mode, 1 missed cleavage was allowed. Percolator [58] was used to assess peptide and protein identification confidence: Protein identifications contain at least two peptides matches that meet or exceed the threshold values for 99% confidence level. The ion score is -10*Log (P), where P is the probability that the observed match is a random event. Peptide identification was performed manually. After database searching, a set of peptide matches was ranked according to their corresponding Mascot scores.

### ESI-Quadrupole-Orbitrap quantification

For the quantification of the trypsinized peptides of inflow and outflow segments of three dogfish specimens we used an ESI-Orbitrap-Quadrupole system. The Quadrupole-Orbitrap (Q Exactive Plus Thermo Scientific, Waltham, MA, USA) was coupled online through a nanoESI emitter (10 μm tip; New Objective; Woburn, MA, USA) using a FlexIon nanospray apparatus (Thermo Scientific, Waltham, MA, USA). Data were acquired using XCalibur v3.0 in data-dependent acquisition mode, using a Top20 method. Survey mass range was 300−1750 m/z. The quadrupole isolation window was set to 1.5 m/z; the AGC target was set to 1 × 106, a maximum injection time of 120 ms, and a resolution of 70000. In MS2 scans, target AGC was set to 5 × 105 with a maximum injection time of 60 ms. Protein quantification was achieved by the Label Free Quantification method, using the results from three different samples.

### A4.1025 immunoprecipitation and affinity analysis

To estimate the relative affinity of each MyHC isoform for the antibody A4.1025 we conducted an immunoprecipitation assay, using one of the extracts previously subjected to ESI-Quadrupole-Orbitrap quantification. For the immunoprecipitation, A4.1025-coated magnetic microbeads (Miltenyi Biotec, Auburn, CA) were used according to the manufacturer instructions. Quantification of the proteins from the eluate was performed by ESI-Orbitrap-Quadrupole as we previously described. In order to calculate the relative affinity of the four MyHC isoform identified in the previous proteomic analyses, we used extracts from the outflow segments, in which the four sarcomeric MyHC isoforms are significantly represented. The relative affinity of each isomyosin was calculated by dividing the concentration of each isoform in the eluate by the initial concentration of the isoform in the same extract.

## Data Availability

The datasets analysed during the current study are available in the UniProtKB/Swiss-Prot repository, https://www.uniprot.org/.

## Abbreviations

ACN: Acetonitrile
BSA: Bovine serum albumin
DTT: Dithiothreitol
FA: Formic acid
HPLC-ESI-MS/MS: High-performance liquid chromatography-electrospray ionisation tandem mass spectrometry
MLC: Myosin Light Chain
MS: Mass spectrometry
MyHC: Myosin Heavy Chain
MyoMiR: Skeletal muscle microRNA
PBS: Phosphate buffer saline
SBT: 1% bovine serum albumin (BSA) and 0.1% Triton X-100 in TPBS
SDS-PAGE: Sodium dodecyl sulfate polyacrylamide gel electrophoresis
TPBS: Tris- Phosphate buffer saline
